# Stress, Professional Lifestyle, and Telomere Biology in Elite Athletes: A Growing Trend in Psychophysiology of Sport

**DOI:** 10.3389/fpsyg.2020.567214

**Published:** 2020-11-04

**Authors:** Amir Hossien Mehrsafar, Miguel Angel Serrano Rosa, Ali Moghadam Zadeh, Parisa Gazerani

**Affiliations:** ^1^Department of Sport Psychology, Faculty of Sports Sciences, University of Tehran, Tehran, Iran; ^2^Department of Psychobiology, Faculty of Psychology, University of Valencia, Valencia, Spain; ^3^Laboratory of Cognitive and Affective Neuroscience, Faculty of Psychology, University of Valencia, Valencia, Spain; ^4^Department of Psychology, Faculty of Psychology and Education, University of Tehran, Tehran, Iran; ^5^Department of Health Science and Technology, Faculty of Medicine, Aalborg University, Aalborg, Denmark

**Keywords:** telomere, telomerase, competition, stress, elite athletes

## Abstract

Professional lifestyle and championship period often put a great deal of pressure on athletes, who usually experience highly stressful periods during training for competitions. Recently, biomarkers of cellular aging, telomere length (TL) and telomerase activity (TA), have been considered to investigate the effects of stress and lifestyle factors. Studies in non-athletic populations have shown that stress and poor lifestyle decrease TL and TA. On the other hand, it has been shown that in general, exercise increases TL and its activity, although the underlying mechanisms remained largely unexplored. TL and TA outcomes in elite athletes remain inconclusive and mainly affected by confounding factors, such as age. Elite athletes, therefore, might offer a unique target group for studying exercise-telomere hypothesis for further investigation of the roles of stressors on telomere-related biomarkers. In this perspective, we highlight the potentials for studying these psychophysiological markers in elite athletes in order to understand stress-aging relationship and potential underlying mechanisms. Moreover, we present important methodological aspects that could help in the development of future experimental designs.

## Introduction

High demand training plans, following precise dietary programs, and attending a large number of competitions, often pressurize athletes, both physically and mentally. Stress is an inevitable factor and a common feature in competitive sports events, and there is no doubt that elite athletes undergo higher demands and are required to overcome these challenges for a successful preparation, performance, and competition (Campbell et al., 2018). Even though coaches and trainers try to adjust the athletes’ training loads, intensive plans for engagement of athletes in training programs are documented in the literature (Pope et al., 2018). For instance, some athletes undergo 15–20 h of intensified training per week for years, which often results in an inadequate recovery time. Recovery is, however, highly important to diminish the risk of injuries and “overtraining syndrome” in elite athletes (Ehrlenspiel and Strahler, 2012). Overloading and overtraining can consequently cause long term and damaging physiological (e.g., decreased the testosterone/cortisol ratio) and psychological (e.g., burnout) effects (Freitas et al., 2014).

Several types of stress have been identified that play a role in overloading elite athletes prior to, during, and after competitions. Those include mental, physical, and technical demands for adequate preparation before competitions, such as demanding training environments, stressful coaching attitude, family stresses, imbalance between sport and non-sport lifestyle, and unrealistic commitments or expectations (Hanton et al., 2005). During or after competitions, other stressors play similar roles, such as rivalry, satisfying the expectations (e.g., media, fan, professional organizations, e.g., better ranking, or dealing with a diverse range of consequences following a non-successful competition) (Wilding, 2014). Collectively, various stressors exist and hence it is important to identify and apply strategies for minimizing or coping with those, which are highly individualized, mainly depending on personal capacity and available resources to each elite athlete. Acute stress and overcoming those seem feasible in many cases; however, chronic stress is often challenging to deal with and can cause long-term psychological and physical damages (Mariotti, 2015; Sabato et al., 2016). Some athletes, for instance, might not be able to apply adaptive coping strategies, which may mitigate the impacts of an innately stressful environment, and those are at higher risks for developing tissue injury and mental disorders, such as depression and anxiety (Purcell et al., 2019). These reactions are often due to activation of other cascades following the chronic activation of systems contributed in the stress response. For instance, overproduction of hormones, may lead to impaired metabolism and immune system that consequently influence overall well-being, performance and behavior of athletes (Lovallo and Buchanan, 2017). Several longitudinal investigations have shown that anxiety and stress as well as poor lifestyle are among important risk factors for a number of physical conditions, including diabetes, coronary heart disease, neurodegenerative, and autoimmune disorders, along with an increase rate of cancer and mortality (Seib et al., 2014). However, epidemiological studies and systematic reviews reported that elite athletes appear with more longevity and slightly lower mortality rate (with standard mortality ratio) compared with the general population (Kettunen et al., 2015; Lemez and Baker, 2015; Antero et al., 2020). In addition, physical fitness in elite athletes has been related to lower risks of somatic diseases (Teramoto and Bungum, 2010).

Accumulating evidence from the past decade suggests that one of the pathways through which stress may impact health is through accelerated cell aging as indexed by the length of the telomeric DNA at the end of chromosomes (Puterman et al., 2010). As a result, telomere length (TL) has emerged as a widely recognized biomarker of biological age. Short TL has been linked to a range of health problems, poor lifestyle, and early mortality (Epel et al., 2006). In this regard, the literature presents that there is a link between stress and shorter TL. Although no studies have yet identified potential moderators of this relationship, several studies have examined health behaviors as potential mediators through which stress affects health (O’Donovan et al., 2012; Shalev et al., 2013). This field has also captured high attention among sport scientists. The scientific literature suggests that a specific health behavior, such as physical activity, can moderate the impact of stress on cell aging (Puterman et al., 2018). Recent studies demonstrate that maintenance of a physically active lifestyle is related to longer TL. It is hypothesized that one mechanism of exercise-associated telomere lengthening is through increased levels of telomerase activity (TA) (Puterman et al., 2018). However, other potential mechanisms have also been proposed to describe how exercise may affect TL, including inflammation, oxidative stress, and proliferation or differentiation of satellite cells (Arsenis et al., 2017). Moreover, it has been made clear that elite athletes have longer TL than inactive and non-elite athletes (Abrahin et al., 2019). Although conflicting outcome exists in the literature (Rae et al., 2010), it also remains unclear whether and how the professional lifestyle of elite athletes and the competition-induced stress and anxiety or intensive training would affect cellular aging. Therefore, we emphasize on the potentials for studying these biomarkers in elite athletes in order to understand stress-aging relationship and underlying mechanisms. In this perspective, we first briefly explain the telomere biology and its relation to stress. Second, we review the relationship between mental disorders, psychological variables, lifestyle factors, interventions, and telomere/telomerase dynamics. Finally, we propose an overview from the dynamics of telomere and telomerase in elite athletes and methodological considerations in the measurement of TL and TA.

## Biology of Telomere and Effective Factors

### Functions of Telomere/Telomerase

A telomere is a region at each end of a chromosome that protects the DNA. TL appears to be a marker of physiological age and it is related to several age-related diseases, lifespan, cancer, and lifestyle factors (Jylhävä et al., 2017). Human telomeres consist of tandem 5’-TTAGGG-3’ repeats, and they form a loop-like structure; therefore, the very end regions of telomeres are concealed, and the end of chromosomes would not be identified as double-strand breaks (Blackburn, 2010). Upon shortening a telomere to a crucial length, the loop structures would not be able to be formed. Hence, the resulting telomere would be recognized as a nick in double-strand DNA, through the activation of DNA damage responses, resulting in the induction of cellular aging and programmed cell death (Pickett and Reddel, 2012). Dysfunction of telomeres may also lead to end-to-end fusions or end-degradation, causing genomic instability. Cellular aging and programmed cell death are thought to participate in the process of aging in normal cells, while genomic instability is considered a sign of cancer (Maciejowski and de Lange, 2017). In healthy somatic cells, TL represents a “mitotic clock” that is able to regulate how many divisions a particular cell can undergo. At least two primary mechanisms have been proposed by which the shortening process of telomeres could occur. First, the replication of telomeres possesses a natural “end-replication problem,” during cell division. In other words, the DNA sequences located at the edge of the linear chromosomes are not capable of being entirely replicated by DNA replication machinery. Second, it has been observed that the process of oxidative stress, caused by the overproduction of reactive oxygen species, can explicitly cause breaks at 5’-TTAGGG-3’ repeats, leading to the shortening of the TL (Baird, 2008).

Human telomerase constitutes two significant subunits, a catalytic enzyme human telomerase reverse transcriptase (hTERT) and an RNA template (hTR or hTERC). The telomerase enzyme employs its RNA template for synthesizing TTAGGG sequences to resolve the obstacle of telomere shortening. In addition to the classic function of telomere lengthening, the telomerase enzyme has several other duties that are independent of the TL (so-called extra-telomere activity), such as increasing stress-resistance, cell survival, protection of mitochondrial functions, mediating DNA damage response, inhibition of apoptosis, and promoting neuroprotective signaling (Cong and Shay, 2008). These properties are essential for the anti-aging process. The TA is controlled by post-translational modifications of the hTERT protein, including phosphorylation and nuclear translocation as well as transcriptional control of hTERT (Wojtyla et al., 2011). More precisely, experiments have demonstrated that alterations in the TA might occur within minutes to a few hours following the exposure to specific molecular stimuli, such as inflammatory cytokines, stress hormones, and growth factors leading to post-translational modifications of the hTERT protein (de Punder et al., 2019).

### Stress and Telomere Biology

Acute stress response is regularly referred to as a spectrum of affective, cognitive, behavioral, and physiological responses to specific stressors, formed by basal physiological circumstances and cognitive biases (Epel et al., 2018). The response of this multi-system may include anticipatory arousal prior to a stressful situation, peak reactivity during an event, and recovery to baseline following a stressful event. Inappropriate response to acute stress may result in detrimental changes in telomere regulation. For instance, autonomic over-reactivity has been correlated with decreased immune cell function, as well as increased cortisol reactivity to stressors with shorter length of telomeres within the immune cells (Jiang et al., 2019). Perseverative cognition, e.g., rumination and worriness, are capable of exacerbating the increased physiological reactivity and delayed recovery, and it may serve as internal stressors. Notably, shorter TL has also been associated with perseverative cognition, such as negative mind wandering and more importantly, anticipatory threat appraisals to acute stressors (Conklin et al., 2019).

The profile of acute stress reactivity is mainly affected by allostatic cases, such as basal levels of neuroendocrine and autonomic activity, inflammation, and metabolic hormones. Prolonged reactivity and chronic exposure to a particular stressor may lead to disturbed allostatic states, followed by pernicious health consequences (Goldstein and McEwen, 2002). The regulation of telomeres seems to be involved in this condition, as a lower TA and shorter TL have been linked to the decreased vagal tone, increased basal levels of cortisol, oxidative stress, and inflammation (Conklin et al., 2019).

Collectively, these investigations propose that chronic stress expedites the process of cellular aging (Epel et al., 2004). Several mechanisms underlying the relationship between telomere dynamics and stress have been characterized (Epel et al., 2009). One of such facets is the impaired allostatic load model that proposes the stress can affect the control of the HPA axis, thereby boosting the secretion of cortisol, participating in allostatic load, and in turn dysregulating telomere maintenance. Indeed, in humans, shorter TL is correlated with higher cortisol reactivity, and *in vitro* evidence shows that increased glucocorticoid concentrations are linked with diminished TA (Jiang et al., 2019). Although, some studies have suggested that the testosterone levels were positively associated with TL (Drury et al., 2014); hence, remained the field with contradictory results (especially in TA).

Nonetheless, it is necessary to perceive that under acute stress conditions, circulating immune cells would be depleted. Thus, a compensatory increase occurs to replace the eliminated cells with young ones, resulting in the longer TL determination and higher TA. Alternatively, chronic stress leads to the induction of continued replication stress and in turn, stimulates telomere attrition and reduces TA. This phenomenon explains the “telomerase paradox”: under acute stress conditions, telomerase would be more activated to protect telomeres; while in chronic stress, as observed in depressed individuals, the activity may be lower, leading to progressive telomere dysfunction (Epel, 2012). A stress triad about the maintenance of telomere has been suggested elsewhere (Epel and Prather, 2018). Basically, chronic exposure to stressors leads to continuously higher perceived stress and subsequent stress arousal, which in turn remarkably influence telomere attrition (Mathur et al., 2016). Other reports propose that inflammation plays a crucial role in telomere attrition and that continued stress is associated with the shorter TL and low-grade inflammation compared with healthy conditions without the presence of any inflammation (Squassina et al., 2019).

### Mental Disorders, Psychological Variables, and Telomere/Telomerase Dynamics

In relation to mental disorders, shortened TL and decreased TA have been associated with mood disorders (such as depression and bipolar disorder), schizophrenia and other psychotic disorders, obsessive-compulsive disorder, anxiety disorders (such as post-traumatic stress disorder, panic disorder, social phobia, and generalized anxiety disorder), and psychoactive substance use (see for review, Deng et al., 2016; Boccardi and Boccardi, 2019). It is noteworthy that robust preclinical and clinical evidence suggests that psychotropic medication (e.g., antidepressants) may exert a positive effect on TL and TA in psychiatric disorders (Zhou et al., 2011). In addition, we identified the literature pointing to some psychosocial variables, including higher perceived stress, distress, defensiveness, anxiety scores and poor mental health, socioeconomic status, and social support that have been correlated to the shortened TL and decreased TA (Starkweather et al., 2014). It has been made clear that the positive dispositions characteristics such as optimism, emotional intelligence, and trait mindfulness as well as problem-focused coping styles were associated with longer TL (Schutte et al., 2016; Archer, 2017).

### Lifestyle and Telomere/Telomerase Dynamics

Studies have examined TL and TA in various lifestyle contexts. Our literature review revealed that physical exercise might have a positive effect on TA and TL. Several animal models and experiments in clinical and non-clinical settings on humans have been carried out to study the effect of physical exercise on the TA and TL (Deng et al., 2016; Stellos and Spyridopoulos, 2019). While some systematic reviews (Mundstock et al., 2015; Lin X. et al., 2019) have demonstrated beneficial effects of physical activity on TL and TA, mainly with moderate level of exercise compared with low or intense exercise, other systematic reviews (Ludlow et al., 2013; Arsenis et al., 2017) could not conclude if a relationship exists between physical activity and TL and TA. In particular, it is still debated whether exercise can directly impact TL. Notwithstanding, some studies highlighted that high exercise load has been related to a decrease in the TL and an increase in TA (Bruunsgaard et al., 1999). In this regard, de Carvalho Cunha et al. (2018) showed that more vigorous exercise with above lactate threshold could also decrease the expression of proteins related to telomere protection (p53 activity and sheltering proteins). Moreover, a new animal experiment has shown that high intensity interval training with short- and long-term intervals does not change the TA (Sadeghi-Tabas et al., 2020). Though there are some important methodological shortcomings (e.g., patterns specific to cell-type or genotype, heterogeneity of studies, small sample size, blinding of researchers, etc.) in relation to load of exercise and telomere biology. Researchers have suggested that more effort is required to mechanistically examine the impact of various modalities of exercise on TL and TA, and future studies need to questions about design of exercise modalities, not only exercise type, but also the intensity, method, or type of stimuli (Jiménez-Pavón et al., 2019).

Several studies have also assessed the impact of diet on TL and TA, and suggest that low-calorie restriction, prolonged fasting, and overeating decrease both TL and TA (Deng et al., 2016). Consumption of meals high in fiber and vitamins (both dietary and supplemental) is related to telomere regulation (higher TL), whereas eating processed meats and foods high in polyunsaturated fats is related to shorter TL. In a multiethnic study, researchers have identified that higher intake of processed meat is significantly associated with shorter TL (Nettleton et al., 2008). The role of sleep in telomere/telomerase dynamics has been described well. Previous studies reported that poor sleep quality and sleep less than 6 h per night have been correlated with shorter TL and lower TA (Shalev et al., 2013). Moreover, excessive alcohol consumption, and cigarette smoking and tobacco consumption have also been associated with shorter TL and TA (Weischer et al., 2014). Collectively, these findings present that diet and lifestyle, and habits can markedly influence both TL and TA. Since athletes follow a special diet or follow specific life styles, it is important to consider when investigating TL and TA in this population compared with non-athlete matched individuals.

### Psycho-Behavioral Interventions and Telomere/Telomerase Dynamics

It is important to note that some lifestyle and psycho-behavioral interventions, including mindfulness, yoga, qigong practice intervention, cognitive behavioral trophies interventions, and meditation could influence TL and TA under healthy and pathological conditions (Schutte and Malouff, 2014; Deng et al., 2016; Conklin et al., 2019). A pioneering study on the effect of an integrative health promotion intervention, including low-calories foods, moderate aerobic exercise, psycho-behavioral practice, and group support session, has observed that TA increased significantly after the 3-month intervention where it was also significantly correlated with decreases in psychological distress (Ornish et al., 2013). In this regard, it is still not clear which interventions might produce the optimal effect on TL and TA.

### Telomere/Telomerase Dynamics in Elite Athletes

Investigations on telomere/telomerase dynamics in elite athletes are limited; however, a growing body of empirical research has shown that young elite athletes have longer TL compared with their inactive peers (Muniesa et al., 2017). Moreover, a group of researchers reported that the whole blood leukocyte telomeres were longer in elite endurance athletes compared with healthy controls (Sousa et al., 2020). In addition, Simoes et al. (2017) indicated that elite sprinters had longer TL, lower body fat and BMI, and a better lipid profile than age-matched controls. Noteworthy, a study in eight professional marathon runners indicated that TA in peripheral blood leukocytes before and after running seven marathons in 7 days did not significantly differ, demonstrating that the impact of physical activity on TA may become saturated in individuals involved in elite endurance athletic activities (Laye et al., 2012). On the other hand, Werner et al. (2009) have demonstrated that in peripheral blood leukocytes, isolated from professional endurance athletes TA, expression of telomere-stabilizing proteins, and downregulation of cell-cycle inhibitors have been increased compared with untrained individuals. A new meta-analysis has concluded that high level chronic physical training (aerobic and resistance training) may provide protective effects on TL (Abrahin et al., 2019). However, one needs to consider the influence of variables in a diverse range of studies that can alter the outcome of TL. For example, elite athletes are motivated to choose difficult lifestyles and frequent delivery of stress in the competition and the championship period that cause a higher risk of injury or illness. This in turn may negatively impact their health and faster aging in some periods or overall in the life span (Tanaka and Seals, 2008). If this hypothesis turns out to be correct, lifestyle associated with their needs for rigorous training-competition and dietary requirements could be chosen in such a way to modulate markers of chronic inflammation and redox balance, to yield a healthier functional aging and athletic performance (Mikkelsen et al., 2013).

## Methodological Considerations for TL and TA Measurement

With a growing research interest in telomere biology, a consensus in laboratory measurements seems critical with high precision and accuracy. Currently, the TL and TA are measured in many different laboratories utilizing different assays (e.g., telomere restriction fragment, length analysis by Southern blot analysis, quantitative PCR, and Telomerase Repeat Amplification Protocol) (Epel et al., 2010; Lin J. et al., 2019). Application of different approaches makes it challenging to compare results from different studies.

Based on tissue type and collection methods (e.g., blood including plasma, serum and peripheral blood mononuclear cells and saliva samples, including swabs and buccal cells), several specimen types have been used for TL and TA measurement (Lin J. et al., 2019). Each specimen offers advantages and challenges and, due to cell type differences, it might influence TL and TA outcomes. For instance, quantitative-PCR provides the advantage of being able to use smaller amounts of DNA, thereby making it amenable to epidemiology studies involving large numbers of people. An alternative method uses fluorescent probes to quantify not only mean TL, but also chromosome-specific TL. Of note, all these novel techniques for TA measurement are currently at the proof of concept stage, and only a number of those have been applied in studies involving clinical tissues or body fluid samples. When incorporating TL and TA into a research study, it is important to thoroughly evaluate the research question, population, sample type, timing of the analysis, and available resources in order to select optimal TL and TA measurement method.

To our knowledge, no study has evaluated TA and TL in elite athletes within the sport context, such as competition (prior, during and after). In future studies, measuring TA and TL biomarkers in this population may require extra attention in methodology with a rigorous design to accommodate specificity and characteristics of this population.

## Conclusion and Future Directions

**FIGURE 1:**
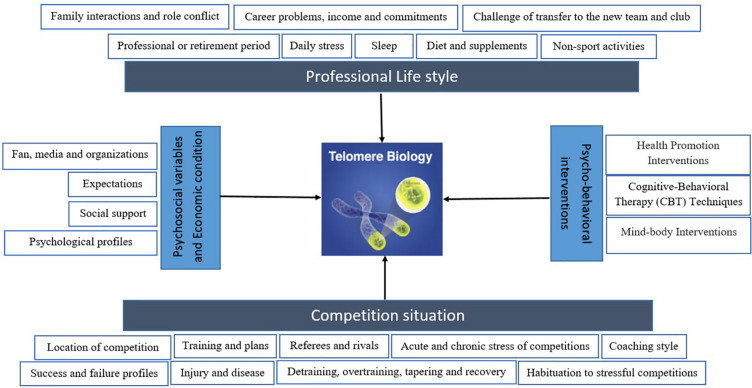
A schematic model depicting competition and its effective factors as well as professional lifestyle that may effect on telomere biology in elite athletes. Moreover, psychosocial variables and economic condition can alter telomere length (TL) and telomerase activity (TA). Additionally, there are multiple psycho-behavioral intervention that may result in the TL and TA changes, including increases in intracellular TA and lengthening of telomeres. The biological mechanisms must be considered to outline a map of a more complete model.

In general, studies in different populations have shown that lifestyle together with acute and chronic stress affect cellular aging. Psychological disorders, interventions, physical activity, diet, sleep, alcohol consumption, and smoking may alter cellular aging differently and through a diverse range of mechanisms that are currently under investigation. Target population is important in this context and elite athletes have been less studied, while this population can offer a unique population for studying biomarkers of aging, including TL and TA. Few studies are available in elite athletes, but those have mainly focused on physiological aspects, and a lack is evident in relation to psychological and lifestyle factors influencing TL, and TA in this population. Figure 1 depicts a simplified overview of potential parameters and aspects that can influence telomere biology in athletes, within sport science and athletic competitions.

Moreover, psychosocial variables and economic condition can alter TL and TA. Additionally, there are multiple psycho-behavioral interventions that may result in the TL and TA changes, including increases in intracellular TA and lengthening of telomeres. The biological mechanisms must be considered to outline a map of a more complete model.

There are a number of open questions that investigators are encouraged to pay attention to for the future studies in this regard. One of the critical points is to consider the level of competition (e.g., long-term league as a chronic stress and tournaments as well as playoff matches as acute stress) and the level of competitive stress on the dynamics of telomere/telomerase. For instance, the greater importance of competition (e.g., final and pre-final competitions) causes more drastic changes in the level of salivary stress markers (Chennaoui et al., 2016). On the other hand, long-term league may suppress immunity function and increase the risk of physical injury in elite athletes (Papacosta et al., 2013).

Psychological variables (e.g., perceived stress, stress reactivity, trait anxiety, coping, etc.) potentially influencing telomere/telomerase are not well investigated either. Since these variables have been well-documented in non-sport literature, and populations with psychological disorders and healthy individuals, can also inspire elite sport studies in the future. Considering that a few elite athletes may also have a range of psychological disorders (Purcell et al., 2019), this sub-population might also offer a platform for investigation of psychological factors. On this line, the International Olympic Committee has focused on the identification, diagnosis, signs, and symptoms, as well as the treatment of these disorders. Studies have demonstrated that psychological interventions, such as meditation and mindfulness, are capable of increasing the TL and TA in clinical populations and patients (Schutte and Malouff, 2014). Future interventions in the field of competitive stress can examine this hypothesis. Stress prevention, management techniques, and changing the stress mindset of elite athletes (even in the adolescence when they are deciding to dedicate their life to be professional), as well as offering sport psychology services to elite athletes for better management of competitive stress and life interactions can be a new perspective in telomere biology in professional athletes.

The type sport and competition, competition season periodization, and intensity of exercise, along with considering gender and age, can increase the generalizability of future studies. In this regard, recent reviews and observational experiences (Ehrlenspiel and Strahler, 2012; Slimani et al., 2017; van Paridon et al., 2017) have indicated that some variables are associated with the psychophysiological changes to competition in elite athletes (e.g., challenge of transfer to the new team and club, location of competition, habituation to stressful competitions, poor sleep, referee and rivals, gender, type of sport, social interaction with fans, media and organizations, warm-up and preparing for competition, coaching styles, social support, expectations, preparation levels, success and failure profiles, commitments and plans, etc.). Each of these variables is worth investigating in the field of telomere biology in future studies. Some of the essential questions that need to be investigated are related to detraining, overtraining, tapering, and recovery periods in elite athletes. Moreover, it is not yet clear if training and exercise might be highly effective to overcome TL shortening and this makes the complex puzzle of mechanisms a subject for further investigation.

The effects of doping and even placebo effects of doping on these cellular aging markers are still opaque. On this subject, an animal model has been reported, in which Stanozolol (a performance-enhancing anabolic androgenic steroid) could induce TA in the liver tissue of rats and exercise reversed this induction, reflecting possible premature aging in the liver tissue (Ozcagli et al., 2018). This area could be a hot topic for future research and provide recommendations to the World Anti-Doping Agency. Physical injuries are one of the areas of interest in telomere biology. It has been made clear that the relative TL in patients (anterior cruciate ligament rupture) with non-contact sports was greater than those with contact sports (Daechavijit et al., 2019). Furthermore, injuries and psychological indices associated with a sports injury, for example, injury, anxiety, and returning to competition after the injury, can open a new horizon in this field.

Scientific research has unraveled the impact of lifestyle and its influencing factors, such as daily stress, family interaction, sleep parameters, diet, physical activity, and smoking on telomere/telomerase dynamics in different populations (Weischer et al., 2014; Deng et al., 2016). Future studies should provide the opportunity to study the lifestyle indicators in elite athletes. For instance, an elite athlete may practice long hours along with external non-sport activities and education, and be subject to overtraining and burnout. Interactions within the family (e.g., spouse) and parents can also be effective indicators. Moreover, the retirement period of elite athletes can offer a potential for studying aging of this population compared with non-competitor athletes. Investigations have estimated that the retirement period (especially in involuntary retirement) and end of athletic career steps sometimes accompanied by sickness, role conflict, loneliness, economic damage, addiction, reduced social support, and depression (Wylleman et al., 2015; Mannes et al., 2019). Looking at potential interventions (e.g., regular exercise and psychology-medical services) in retirement period can be helpful for addressing potential mechanisms of TL and TA.

Since blood sampling and complicated measurement techniques might be challenging during a competitive situation, non-invasive methods, for example, obtaining salivary samples, would be beneficial. Some recommendations have been created (Lin J. et al., 2019) that are continually being updated. In addition, to find the mechanisms of TL and TA changes, investigation of intracellular cascades (not only *in vivo* but also *in vitro*) must be considered.

Taken together, in the context of elite athletes involved in highly competitive sports, several psychological, neurological, hormonal, immunological, oxidative, and cellular responses play roles in aging that are not yet thoroughly investigated. The longitudinal studies are warranted to investigate the possible underlying mechanisms of the effects of lifestyle, competition-induced stress, and athletes’ championships period on cellular markers of aging to identify if a particular dynamic affects TL and TA in this population. This would in turn result in identification of modifiable factors, such as lifestyle changes, or dietary recommendations for elite athletes to experience a healthier life and aging.

## Author Contributions

All the authors discussed the hypothesis and the manuscript content, wrote the first draft, and read and approved the final manuscript.

## Conflict of Interest

The authors declare that the research was conducted in the absence of any commercial or financial relationships that could be construed as a potential conflict of interest.

## References

[B1] Abrahin O. Cortinhas-Alves E. A. Vieira R. P. Guerreiro J. F. (2019). Elite athletes have longer telomeres than sedentary subjects: a meta-analysis. *Exp. Gerontol.* 119 138–145. 10.1016/j.exger.2019.01.023 30735724

[B2] Antero J. Tanaka H. De Larochelambert Q. Pohar-Perme M. Toussaint J.-F. (2020). Female and male US Olympic athletes live 5 years longer than their general population counterparts: a study of 8124 former US Olympians. *Br. J. Sports Med.* 10.1136/bjsports-2019-101696 [Epub ahead of print]. 32727712

[B3] Archer T. (2017). Health benefits for ageing: positive affect and life satisfaction, exercise and coping, and telomere length. *J. Ment. Health Aging* 1 13–17.

[B4] Arsenis N. C. You T. Ogawa E. F. Tinsley G. M. Zuo L. (2017). Physical activity and telomere length: impact of aging and potential mechanisms of action. *Oncotarget* 8 45008–45019. 10.18632/oncotarget.16726 28410238 PMC5546536

[B5] Baird D. M. (2008). Telomere dynamics in human cells. *Biochimie* 90 116–121. 10.1016/j.biochi.2007.08.003 17854970

[B6] Blackburn E. H. (2010). Telomeres and telomerase: the means to the end (Nobel lecture). *Angew. Chem. Int. Ed.* 49 7405–7421. 10.1002/anie.201002387 20821774

[B7] Boccardi M. Boccardi V. (2019). Psychological wellbeing and healthy aging: focus on telomeres. *Geriatrics* 4:25. 10.3390/geriatrics4010025 31023993 PMC6473912

[B8] Bruunsgaard H. Jensen M. S. Schjerling P. Halkjær-Kristensen J. Ogawa K. Skinhøj P. (1999). Exercise induces recruitment of lymphocytes with an activated phenotype and short telomeres in young and elderly humans. *Life Sci.* 65 2623–2633. 10.1016/S0024-3205(99)00531-7 10619370

[B9] Campbell E. Irving R. Bailey J. Dilworth L. Abel W. (2018). Overview of Psychophysiological Stress and the Implications for Junior Athletes. *Am. J. Sports Sci. Med.* 6 72–78.

[B10] Chennaoui M. Bougard C. Drogou C. Langrume C. Miller C. Gomez-Merino D. (2016). Stress biomarkers, mood states, and sleep during a major competition:“Success” and “failure” athlete’s profile of high-level swimmers. *Front. Physiol.* 7:94. 10.3389/fphys.2016.00094 27014092 PMC4789459

[B11] Cong Y. Shay J. W. (2008). Actions of human telomerase beyond telomeres. *Cell Res.* 18 725–732. 10.1038/cr.2008.74 18574498

[B12] Conklin Q. A. Crosswell A. D. Saron C. D. Epel E. S. (2019). Meditation, stress processes, and telomere biology. *Curr. Opin. Psychol.* 28 92–101. 10.1016/j.copsyc.2018.11.009 30553080 PMC6526075

[B13] Daechavijit P. Siridonthanakasem J. Wongsupha P. Yuktanandana P. Honsawek S. (2019). Relative telomere length in blood leukocytes of patients with anterior cruciate ligament injury: a pilot study. *Malaysian Orthopaedic J.* 13 8–13. 10.5704/MOJ.1903.001 31001377 PMC6459039

[B14] de Carvalho Cunha V. N. dos Santos Rosa T. Sales M. M. Sousa C. V. da Silva, Aguiar S. (2018). Training performed above lactate threshold decreases p53 and Shelterin Expression in Mice. *Int. J. Sports Med.* 39 704–711. 10.1055/a-0631-3441 29945271

[B15] de Punder K. Heim C. Wadhwa P. D. Entringer S. (2019). Stress and immunosenescence: the role of telomerase. *Psychoneuroendocrinology* 101 87–100. 10.1016/j.psyneuen.2018.10.019 30445409 PMC6458519

[B16] Deng W. Cheung S. T. Tsao S. W. Wang X. M. Tiwari A. F. Y. (2016). Telomerase activity and its association with psychological stress, mental disorders, lifestyle factors and interventions: a systematic review. *Psychoneuroendocrinology* 64 150–163. 10.1016/j.psyneuen.2015.11.017 26677763

[B17] Drury S. S. Shachet A. Brett Z. H. Wren M. Esteves K. Shirtcliff E. A. (2014). Growing up or growing old? Cellular aging linked with testosterone reactivity to stress in youth. *Am. J. Med. Sci.* 348 92–100. 10.1097/MAJ.0000000000000299 25010187 PMC4122251

[B18] Ehrlenspiel F. Strahler K. (2012). *Psychoneuroendocrinology of Sport and Exercise: Foundations, Markers, Trends.* Abingdon: Routledge 10.4324/9780203133743

[B19] Epel E. (2012). How “reversible” is telomeric aging? *Cancer Prev. Res.* 5 1163–1168. 10.1158/1940-6207.CAPR-12-0370 23041472

[B20] Epel E. Daubenmier J. Moskowitz J. T. Folkman S. Blackburn E. (2009). Can meditation slow rate of cellular aging? Cognitive stress, mindfulness, and telomeres. *Ann. N. Y. Acad. Sci.* 1172 34–53. 10.1111/j.1749-6632.2009.04414.x 19735238 PMC3057175

[B21] Epel E. S. Blackburn E. H. Lin J. Dhabhar F. S. Adler N. E. Morrow J. D. (2004). Accelerated telomere shortening in response to life stress. *Proc. Natl. Acad. Sci. U.S.A.* 101 17312–17315. 10.1073/pnas.0407162101 15574496 PMC534658

[B22] Epel E. S. Crosswell A. D. Mayer S. E. Prather A. A. Slavich G. M. Puterman E. (2018). More than a feeling: a unified view of stress measurement for population science. *Front. Neuroendocrinol.* 49 146–169. 10.1016/j.yfrne.2018.03.001 29551356 PMC6345505

[B23] Epel E. S. Lin J. Dhabhar F. S. Wolkowitz O. M. Puterman E. Karan L. (2010). Dynamics of telomerase activity in response to acute psychological stress. *Brain Behav. Immun.* 24 531–539. 10.1016/j.bbi.2009.11.018 20018236 PMC2856774

[B24] Epel E. S. Lin J. Wilhelm F. H. Wolkowitz O. M. Cawthon R. Adler N. E. (2006). Cell aging in relation to stress arousal and cardiovascular disease risk factors. *Psychoneuroendocrinology* 31 277–287. 10.1016/j.psyneuen.2005.08.011 16298085

[B25] Epel E. S. Prather A. A. (2018). Stress, telomeres, and psychopathology: toward a deeper understanding of a triad of early aging. *Annu. Rev. Clin. Psychol.* 14 371–397. 10.1146/annurev-clinpsy-032816-045054 29494257 PMC7039047

[B26] Freitas C. G. Aoki M. S. Franciscon C. A. Arruda A. F. Carling C. Moreira A. (2014). Psychophysiological responses to overloading and tapering phases in elite young soccer players. *Pediatr. Exerc. Sci.* 26 195–202. 10.1123/pes.2013-0094 24722819

[B27] Goldstein D. S. McEwen B. (2002). Allostasis, homeostats, and the nature of stress. *Stress* 5 55–58. 10.1080/102538902900012345 12171767

[B28] Hanton S. Fletcher D. Coughlan G. (2005). Stress in elite sport performers: a comparative study of competitive and organizational stressors. *J. Sports Sci.* 23 1129–1141. 10.1080/02640410500131480 16194989

[B29] Jiang Y. Da W. Qiao S. Zhang Q. Li X. Ivey G. (2019). Basal cortisol, cortisol reactivity, and telomere length: a systematic review and meta-analysis. *Psychoneuroendocrinology* 103 163–172. 10.1016/j.psyneuen.2019.01.022 30695740 PMC6450740

[B30] Jiménez-Pavón D. Carbonell-Baeza A. Lavie C. J. (2019). Are changes in telomerase activity and telomere length due to different exercise modalities, intensity, or methods: intermittency? *Eur. Heart J.* 40 3198–3199. 10.1093/eurheartj/ehz323 31132084

[B31] Jylhävä J. Pedersen N. L. Hägg S. (2017). Biological age predictors. *EBioMedicine* 21 29–36. 10.1016/j.ebiom.2017.03.046 28396265 PMC5514388

[B32] Kettunen J. A. Kujala U. M. Kaprio J. Bäckmand H. Peltonen M. Eriksson J. G. (2015). All-cause and disease-specific mortality among male, former elite athletes: an average 50-year follow-up. *Br. J. Sports Med.* 49 893–897. 10.1136/bjsports-2013-093347 25183628

[B33] Laye M. J. Solomon T. P. Karstoft K. Pedersen K. K. Nielsen S. D. Pedersen B. K. (2012). Increased shelterin mRNA expression in peripheral blood mononuclear cells and skeletal muscle following an ultra-long-distance running event. *J. Appl. Physiol.* 112 773–781. 10.1152/japplphysiol.00997.2011 22162529

[B34] Lemez S. Baker J. (2015). Do elite athletes live longer? A systematic review of mortality and longevity in elite athletes. *Sports Med. Open* 1:16. 10.1186/s40798-015-0024-x 26301178 PMC4534511

[B35] Lin J. Smith D. L. Esteves K. Drury S. (2019). Telomere length measurement by qPCR – Summary of critical factors and recommendations for assay design. *Psychoneuroendocrinology* 99 271–278. 10.1016/j.psyneuen.2018.10.005 30343983 PMC6363640

[B36] Lin X. Zhou J. Dong B. (2019). Effect of different levels of exercise on telomere length: a systematic review and meta-analysis. *J. Rehabil. Med.* 51 473–478. 10.2340/16501977-2560 31093683

[B37] Lovallo W. Buchanan T. (2017). “Stress hormones in psychophysiological research: emotional, behavioral, and cognitive implications,” in *Handbook of Psychophysiology (Cambridge Handbooks in Psychology)*, eds Cacioppo J. Tassinary L. Berntson G. (Cambridge: Cambridge University Press), 465–494. 10.1017/9781107415782.021

[B38] Ludlow A. T. Ludlow L. W. Roth S. M. (2013). Do telomeres adapt to physiological stress? Exploring the effect of exercise on telomere length and telomere-related proteins. *Biomed Res. Int.* 2013:601368. 10.1155/2013/601368 24455708 PMC3884693

[B39] Maciejowski J. de Lange T. (2017). Telomeres in cancer: tumour suppression and genome instability. *Nat. Rev. Mol. Cell Biol.* 18 175–186. 10.1038/nrm.2016.171 28096526 PMC5589191

[B40] Mannes Z. L. Waxenberg L. B. Cottler L. B. Perlstein W. M. Burrell L. E. II Ferguson E. G. (2019). Prevalence and correlates of psychological distress among retired elite athletes: a systematic review. *Int. Rev. Sport Exerc. Psychol.* 12 265–294. 10.1080/1750984X.2018.1469162 31217807 PMC6583001

[B41] Mariotti A. (2015). The effects of chronic stress on health: new insights into the molecular mechanisms of brain–body communication. *Future Sci. OA* 1:FSO23. 10.4155/fso.15.21 28031896 PMC5137920

[B42] Mathur M. B. Epel E. Kind S. Desai M. Parks C. G. Sandler D. P. (2016). Perceived stress and telomere length: a systematic review, meta-analysis, and methodologic considerations for advancing the field. *Brain Behav. Immun.* 54 158–169. 26853993 10.1016/j.bbi.2016.02.002 PMC5590630

[B43] Mikkelsen U. R. Couppé C. Karlsen A. Grosset J. F. Schjerling P. Mackey A. L. (2013). Life-long endurance exercise in humans: circulating levels of inflammatory markers and leg muscle size. *Mech. Ageing Dev.* 134 531–540. 10.1016/j.mad.2013.11.004 24287006

[B44] Mundstock E. Zatti H. Louzada F. M. Oliveira S. G. Guma F. T. Paris M. M. (2015). Effects of physical activity in telomere length: systematic review and meta-analysis. *Ageing Res. Rev.* 22 72–80. 25956165 10.1016/j.arr.2015.02.004

[B45] Muniesa C. A. Verde Z. Diaz-Ureña G. Santiago C. Gutiérrez F. Díaz E. (2017). Telomere Length in Elite Athletes. *Int. J. Sports Physiol. Perform.* 12 994–996. 10.1123/ijspp.2016-0471 27918657

[B46] Nettleton J. A. Diez-Roux A. Jenny N. S. Fitzpatrick A. L. Jacobs D. R. Jr. (2008). Dietary patterns, food groups, and TL in the Multi-Ethnic Study of Atherosclerosis (MESA). *Am. J. Clin. Nutr*. 88 1405–1412. 10.1093/ajcn/88.1.185 18996878 PMC3037593

[B47] O’Donovan A. Tomiyama A. J. Lin J. Puterman E. Adler N. E. Kemeny M. (2012). Stress appraisals and cellular aging: a key role for anticipatory threat in the relationship between psychological stress and telomere length. *Brain Behav. Immun.* 26 573–579. 10.1016/j.bbi.2012.01.007 22293459 PMC3322317

[B48] Ornish D. Lin J. Chan J. M. Epel E. Kemp C. Weidner G. (2013). Effect of comprehensive lifestyle changes on telomerase activity and telomere length in men with biopsy-proven low-risk prostate cancer: 5-year follow-up of a descriptive pilot study. *Lancet Oncol.* 14 1112–1120. 10.1016/S1470-2045(13)70366-8 24051140

[B49] Ozcagli E. Kara M. Kotil T. Fragkiadaki P. Tzatzarakis M. N. Tsitsimpikou C. (2018). Stanozolol administration combined with exercise leads to decreased telomerase activity possibly associated with liver aging. *Int. J. Mol. Med.* 42 405–413. 10.3892/ijmm.2018.3644 29717770 PMC5979936

[B50] Papacosta E. Gleeson M. Nassis G. P. (2013). Salivary hormones, IgA, and performance during intense training and tapering in judo athletes. *J. Strength Cond. Res.* 27 2569–2580. 10.1519/JSC.0b013e31827fd85c 23249825

[B51] Pickett H. A. Reddel R. R. (2012). The role of telomere trimming in normal telomere length dynamics. *Cell Cycle* 11 1309–1315. 10.4161/cc.19632 22421147

[B52] Pope C. C. Penney D. Smith T. B. (2018). Overtraining and the complexities of coaches’ decision-making: managing elite athletes on the training cusp. *Reflect. Pract.* 19 145–166. 10.1080/14623943.2017.1361923

[B53] Purcell R. Gwyther K. Rice S. M. (2019). Mental health in elite athletes: increased awareness requires an early intervention framework to respond to athlete needs. *Sports Med. Open* 5:46. 10.1186/s40798-019-0220-1 31781988 PMC6883009

[B54] Puterman E. Lin J. Blackburn E. O’donovan A. Adler N. Epel E. (2010). The power of exercise: buffering the effect of chronic stress on telomere length. *PLoS One* 5:e10837. 10.1371/journal.pone.0010837 20520771 PMC2877102

[B55] Puterman E. Weiss J. Lin J. Schilf S. Slusher A. L. Johansen K. L. (2018). Aerobic exercise lengthens telomeres and reduces stress in family caregivers: a randomized controlled trial-Curt Richter Award Paper 2018. *Psychoneuroendocrinology* 98 245–252. 10.1016/j.psyneuen.2018.08.002 30266522

[B56] Rae D. E. Vignaud A. Butler-Browne G. S. Thornell L.-E. Sinclair-Smith C. Derman E. W. (2010). Skeletal muscle telomere length in healthy, experienced, endurance runners. *Eur. J. Appl. Physiol.* 109 323–330. 10.1007/s00421-010-1353-6 20101406

[B57] Sabato T. M. Walch T. J. Caine D. J. (2016). The elite young athlete: strategies to ensure physical and emotional health. *Open Access J. Sports Med.* 7 99–113. 10.2147/OAJSM.S96821 27621677 PMC5012846

[B58] Sadeghi-Tabas S. Saghebjoo M. Sarir H. Hedayati M. (2020). Effects of work/rest interval manipulation of high-intensity interval training and detraining on telomerase activity and p53 levels in cardiac muscle. *Sci. Sports* 35:170 10.1016/j.scispo.2019.06.002

[B59] Schutte N. S. Malouff J. M. (2014). A meta-analytic review of the effects of mindfulness meditation on telomerase activity. *Psychoneuroendocrinology* 42 45–48. 10.1016/j.psyneuen.2013.12.017 24636500

[B60] Schutte N. S. Palanisamy S. K. McFarlane J. R. (2016). The relationship between positive psychological characteristics and longer telomeres. *Psychol. Health* 31 1466–1480. 10.1080/08870446.2016.1226308 27616348

[B61] Seib C. Whiteside E. Humphreys J. Lee K. Thomas P. Chopin L. (2014). A longitudinal study of the impact of chronic psychological stress on health-related quality of life and clinical biomarkers: protocol for the Australian Healthy Aging of Women Study. *BMC Public Health* 14:9. 10.1186/1471-2458-14-9 24400870 PMC3890545

[B62] Shalev I. Entringer S. Wadhwa P. D. Wolkowitz O. M. Puterman E. Lin J. (2013). Stress and telomere biology: a lifespan perspective. *Psychoneuroendocrinology* 38 1835–1842. 23639252 10.1016/j.psyneuen.2013.03.010 PMC3735679

[B63] Simoes H. G. Sousa C. V. dos Santos Rosa T. da Silva Aguiar S. Deus L. A. Rosa E. C. C. C. (2017). Longer telomere length in elite master sprinters: relationship to performance and body composition. *Int. J. Sports Med.* 38 1111–1116. 10.1055/s-0043-120345 29100249

[B64] Slimani M. Baker J. S. Cheour F. Taylor L. Bragazzi N. L. (2017). Steroid hormones and psychological responses to soccer matches: insights from a systematic review and meta-analysis. *PLoS One* 12:e0186100. 10.1371/journal.pone.0186100 29023546 PMC5638322

[B65] Sousa C. V. Aguiar S. S. Deus L. Barbosa L. P. dos Santos P. A. Neves R. V. P. (2020). Faster and healthier: relationship between telomere and performance in master athletes. *Int. J. Sports Med.* 41 339–344. 10.1055/a-1088-5279 32045948

[B66] Squassina A. Pisanu C. Vanni R. (2019). Mood disorders, accelerated aging, and inflammation: Is the link hidden in telomeres? *Cells* 8:52. 10.3390/cells8010052 30650526 PMC6356466

[B67] Starkweather A. R. Alhaeeri A. A. Montpetit A. Brumelle J. Filler K. Montpetit M. (2014). An integrative review of factors associated with telomere length and implications for biobehavioral research. *Nurs. Res.* 63 36–50. 10.1097/NNR.0000000000000009 24335912 PMC4112289

[B68] Stellos K. Spyridopoulos I. (2019). Exercise, telomerase activity, and cardiovascular disease prevention. *Eur. Heart J*. 40, 47–49. 10.1093/eurheartj/ehy70 30496530

[B69] Tanaka H. Seals D. R. (2008). Endurance exercise performance in Masters athletes: age-associated changes and underlying physiological mechanisms. *J. Physiol.* 586 55–63. 10.1113/jphysiol.2007.141879 17717011 PMC2375571

[B70] Teramoto M. Bungum T. J. (2010). Mortality and longevity of elite athletes. *J. Sci. Med. Sport* 13 410–416. 19574095 10.1016/j.jsams.2009.04.010

[B71] van Paridon K. N. Timmis M. A. Nevison C. M. Bristow M. (2017). The anticipatory stress response to sport competition; a systematic review with meta-analysis of cortisol reactivity. *BMJ Open Sport Exerc. Med.* 3:e000261. 10.1136/bmjsem-2017-000261 PMC5604718 29177073

[B72] Weischer M. Bojesen S. E. Nordestgaard B. G. (2014). Telomere shortening unrelated to smoking, body weight, physical activity, and alcohol intake: 4,576 general population individuals with repeat measurements 10 years apart. *PLoS Genet.* 10:e1004191. 10.1371/journal.pgen.1004191 24625632 PMC3953026

[B73] Werner C. Fürster T. Widmann T. Pöss J. Roggia C. Hanhoun M. (2009). Physical exercise prevents cellular senescence in circulating leukocytes and in the vessel wall. *Circulation* 120 2438–2447. 10.1161/CIRCULATIONAHA.109.861005 19948976

[B74] Wilding A. (2014). An exploration of sources of stress in elite adolescent sport: a case study approach. *Int. J. Sport Soc.* 4 69–72.

[B75] Wojtyla A. Gladych M. Rubis B. (2011). Human telomerase activity regulation. *Mol. Biol. Rep.* 38 3339–3349. 21086176 10.1007/s11033-010-0439-x PMC3085100

[B76] Wylleman P. Rosier N. De Knop P. (2015). “Transitional challenges and elite athletes’ mental health,” in *Health and Elite Sport. Is High Performance Sport a Healthy Pursuit*, eds Baker J. Safai P. Fraser-Thomas J. (Oxon: Routledge), 99–116.

[B77] Zhou Q.-G. Hu Y. Wu D.-L. Zhu L.-J. Chen C. Jin X. (2011). Hippocampal telomerase is involved in the modulation of depressive behaviors. *J. Neurosci.* 31 12258–12269. 21865469 10.1523/JNEUROSCI.0805-11.2011 PMC6623221

